# Long-term Outcome of Children Born to Women with Autoimmune Rheumatic Diseases: A Multicentre, Nationwide Study on 299 Randomly Selected Individuals

**DOI:** 10.1007/s12016-021-08857-2

**Published:** 2021-03-16

**Authors:** Laura Andreoli, Cecilia Nalli, Maria Grazia Lazzaroni, Chiara Carini, Francesca Dall’Ara, Rossella Reggia, Marília Rodrigues, Carolina Benigno, Elena Baldissera, Elena Bartoloni, Fabio Basta, Francesca Bellisai, Alessandra Bortoluzzi, Corrado Campochiaro, Francesco Paolo Cantatore, Roberto Caporali, Angela Ceribelli, Cecilia B. Chighizola, Paola Conigliaro, Addolorata Corrado, Maurizio Cutolo, Salvatore D’Angelo, Elena De Stefani, Andrea Doria, Maria Favaro, Colomba Fischetti, Rosario Foti, Armando Gabrielli, Elena Generali, Roberto Gerli, Maria Gerosa, Maddalena Larosa, Armin Maier, Nazzarena Malavolta, Marianna Meroni, Pier Luigi Meroni, Carlomaurizio Montecucco, Marta Mosca, Melissa Padovan, Giuseppe Paolazzi, Giulia Pazzola, Susanna Peccatori, Roberto Perricone, Giorgio Pettiti, Valentina Picerno, Immacolata Prevete, Véronique Ramoni, Nicoletta Romeo, Amelia Ruffatti, Carlo Salvarani, Gian Domenico Sebastiani, Carlo Selmi, Francesca Serale, Luigi Sinigaglia, Chiara Tani, Marica Trevisani, Marta Vadacca, Eleonora Valentini, Guido Valesini, Elisa Visalli, Ester Vivaldelli, Lucia Zuliani, Angela Tincani

**Affiliations:** 1 Department of Clinical and Experimental Sciences, University of Brescia, Viale Europa 11, 25123 Brescia, Italy; 2 Rheumatology and Clinical Immunology Unit, ASST Spedali Civili, Piazzale Spedali Civili 1, 25123 Brescia, Italy; 3 Centro Hospitalar de Leiria (CHL), Hospital de Santo André (HSA), R. de Santo André, 2410-197 Leiria, Portugal; 4 Rheumatology Research Unit, Department of Clinical Medicine and Surgery, University Federico II of Naples, Via Sergio Pansini 5, 80131 Naples, Italy; 5 Unit of Medicine and Clinical Immunology, IRCCS San Raffaele Scientific Institute, Via Olgettina 60, 20132 Milan, Italy; 6 Department of Medicine, Rheumatology Unit, University of Perugia, Piazzale Gambuli 1, 06132 Perugia, Italy; 7 Unit of Allergology, Clinical Immunology and Rheumatology, University Campus Biomedico Rome, Via Álvaro del Portillo, 21, 00128 Rome, Italy; 8 Rheumatology Unit, Policlinico Le Scotte and University of Siena, Via Mario Bracci-Loc.Le Scotte, 53100 Siena, Italy; 9 Rheumatology Unit, Department of Medical Sciences, University of Ferrara and Azienda Ospedaliero-Universitaria S. Anna, Via Aldo Moro 8, 44124 Cona, Ferrara, Italy; 10 Rheumatology Clinic, Department of Medical and Surgical Sciences, University of Foggia, Rione Biccari, 71122 Foggia, Italy; 11 Department of Rheumatology, ASST Istituto Gaetano Pini & CTO, Piazza Cardinale Andrea Ferrari, 1, 20122 Milan, Italy; 12 Department of Clinical Sciences and Community Health, University of Milan, Via Festa del Perdono 7, 20122 Milano, Italy; 13 Division of Rheumatology and Clinical Immunology, Humanitas Research Hospital, Via Manzoni 56Rozzano Milano, 20089 Milan, Italy; 14 Unit of Rheumatology, Istituto Auxologico Italiano, Via Mosè Bianchi, 90, 20149 Milan, Italy; 15 Rheumatology, Allergy and Clinical Immunology, University of Rome Tor Vergata, Via Oxford 81, 00133 Rome, Italy; 16 Research Laboratory and Division of Rheumatology, Postgraduate School of Rheumatology, Department of Internal Medicine, University of Genova, Viale Benedetto XV 16, 16132 Genova, Italy; 17 Rheumatology Institute of Lucania (IReL) – Rheumatology Department of Lucania, San Carlo Hospital of Potenza and Madonna Delle Grazie Hospital of Matera, Via Potito Petrone 1, 85100 Potenza, Italy; 18 Rheumatology Unit, Department of Medicine-DIMED, University of Padua, Via Nicolò Giustiniani, 2, 35128 Padua, Italy; 19 Clinica Medica, Università Politecnica Delle Marche, Via Tronto, 10/a, 60126 Ancona, Italy; 20 Rheumatology Unit, A.O.U. Policlinico Vittorio Emanuele, Via S. Sofia 78, 95123 Catania, Italy; 21 Rheumatology Outpatient Clinic, Internal Medicine Department, Hospital of Bolzano, Via Lorenz Böhler 5, 39100 Bolzano, Italy; 22 Rheumatology Unit, Internal Medicine, Policlinico S.Orsola-Malpighi, Azienda Ospedaliero-Universitaria Di Bologna, Via Pietro Albertoni 15, 40138 Bologna, Italy; 23 Humanitas Gavazzeni, ASL 5 Spezzino, BergamoLa Spezia, Italy; 24 Division of Rheumatology, University of Pavia and Fondazione IRCCS Policlinico San Matteo, Viale Camillo Golgi 19, 27100 Pavia, Italy; 25 Rheumatology Unit, Department of Clinical and Experimental Medicine, University of Pisa, Via Roma, 67, 56126 Pisa, Italy; 26 Department of Rheumatology, S. Chiara Hospital, Largo Medaglie D’oro 9, 38122 Trento, Italy; 27 Rheumatology Unit, Azienda USL-IRCCS of Reggio Emilia, University of Modena and Reggio Emilia, Viale Risorgimento, 80, 42123 Reggio-Emilia, Italy; 28 Unit of Rheumatology, S.Croce E Carle Hospital, Via Michele Coppino, 26, 12100 Cuneo, Italy; 29 Rheumatology Unit, San Camillo Hospital, Circonvallazione Gianicolense, 87, 00152 Rome, Italy; 30 Rheumatology Unit, Department of Internal Medicine and Medical Specialties, La Sapienza University, Piazzale Aldo Moro 5, 00185 Roma, Italy

**Keywords:** Rheumatic diseases, Reproductive issues, Offspring, Neurodevelopmental disorders, Counselling

## Abstract

The concern about the offspring’s health is one of the reasons for a reduced family size of women with rheumatic diseases (RD). Increased risk of autoimmune diseases (AD) and neurodevelopmental disorders (ND) has been reported in children born to patients with RD. Within a nationwide survey about reproductive issues of women with RD, we aimed at exploring the long-term outcome of their children. By surveying 398 patients who received their diagnosis of RD during childbearing age (before the age of 45), information about the offspring were obtained from 230 women who declared to have had children. A total of 148 (64.3%) patients were affected by connective tissue diseases (CTD) and 82 (35.7%) by chronic arthritis. Data on 299 children (156 males, 52.1%; mean age at the time of interview 17.1 ± 9.7 years) were collected. Twelve children (4.0%), who were born to patients with CTD in 75% of the cases, were affected by AD (8 cases of celiac disease). Eleven children had a certified diagnosis of ND (3.6%; 6 cases of learning disabilities); 9 of them were born to mothers with CTD (5 after maternal diagnosis). No association was found between ND and prenatal exposure to either maternal autoantibodies or anti-rheumatic drugs. Absolute numbers of offspring affected by AD and ND were low in a multicentre cohort of Italian women with RD. This information can be helpful for the counselling about reproductive issues, as the health outcomes of the offspring might not be an issue which discourage women with RD from having children.

## Introduction

Rheumatic diseases (RD) are chronic/inflammatory conditions that can affect women of childbearing age. Counselling about reproductive issues should be a key point of the physician–patient communication [1–3]-

The family size of women with RD is reduced as compared with the general population [4]. Patients reported several reasons for having less children than desired or not having children at all, including the fear that the disease or the medications could harm the foetus, or the concern of not being able to take care of the baby, or that the child could develop the same disease as the mother [5].

Studies available about the long-term outcome of children born to mothers with RD have different design and sample size and are focused on specific conditions and maternal diseases [6], such as neurodevelopmental disorders (ND) in children born to mothers with rheumatoid arthritis (RA) and systemic lupus erythematosus (SLE) [7–9]. Autoimmune diseases (AD) rarely arise during infancy; therefore, only studies covering decades of follow-up of children would be informative. The literature is still lacking long-term studies about this issue. As a whole, it is no easy task to counsel a woman with RD about the long-term outcome of her offspring.

This multicentre, nationwide study aimed at collecting data about the long-term health conditions of children born to women with RD in Italy, with particular focus on the development of AD and ND, in order to gain information for the counselling of patients.

## Methods

This study was part of a multicentre, retro-prospective, patient-based study aimed at exploring the unmet needs of women of childbearing age with RD. As previously described [5], patients with a confirmed diagnosis of RD who were in follow-up at 24 centres affiliated with the Italian Society for Rheumatology (SIR) were invited to participate in the study. The study was approved by the ethical committee (EC) of coordinating centre in Brescia (ASST Spedali Civili). Subsequently, it was approved by the local EC at each centre.

The interviews were conducted in September 2015 by means of a self-reported questionnaire that was proposed to 20 non-selected, consecutive patients attending the outpatient clinic of each centre. The questionnaire comprised 65 multiple-choice and 12 open-answer questions covering six main sections: (1) counselling about contraception and pregnancy, (2) knowledge of RD’s implications for reproductive matters, (3) family size and desire for pregnancy, (4) pregnancy outcomes before and after RD diagnosis, (5) patients’ awareness of the use of drugs during pregnancy, (6) children’s follow-up.

This paper focuses on the follow-up of children born to patients with RD, as the other themes have already been the subject of a previous paper [5]. For the purpose of statistical analysis, patients were subdivided into two groups: connective tissues diseases (CTD) and chronic arthritis (CA). The CTD group comprised systemic lupus erythematosus and/or antiphospholipid syndrome (SLE/APS), dermato-polymyositis (DM-PM), undifferentiated connective tissue disease (UCTD), mixed connective tissue disease (MCTD), primary Sjögren’s syndrome (SS), and systemic sclerosis (SSc). The CA group comprised: rheumatoid arthritis (RA) and seronegative spondyloarthropathies (SpA).

Out of 398 patients, 230 (57.8%) had children. Children’s diagnoses of AD and/or ND reported by the mothers were checked through a second round of interview, and only cases certified by a medical specialist were considered.

The chi-square test for categorical data (or Fisher’s exact when appropriate) and Student’s *t*-test for continuous data were used for statistical analysis. Significance was set at a *p* ≤ 0.05.

## Results

Data were collected for 299 children born to 230 patients with a diagnosis of CA (82, 35.7%; 56 RA, 26 SpA; 58 patients with 1 child, 24 patients with 2 children) or CTD (148, 64.3%; 70 SLE/APS, 6 DM-PM, 18 UCTD, 2 MCTD, 13 SS, 39 SSc; 101 patients with 1 child, 47 with 2 children). Children were male in 156 cases (52.2%) and female in 143 cases (47.8%), with a mean age of 17.1 years (± 9.7) at the time of interview. Fifty-six (18.7%) children were born before the 37th week of gestation; premature birth before the 34th week occurred in 17 cases (5.6%). One case of Trisomy 21 and one perinatal death due to respiratory distress were reported.

No significant differences were observed in the developmental milestones (age at sitting position, walking, first speech, discontinuation of diaper) between children born from mothers with CA or CTD and born before or after maternal diagnosis. Sleep disturbances (pavor nocturnus, somnambulism, insomnia) and feeding problems (food allergy/intolerance) were observed in less than 10% of children.

Regarding school performance, 12 children (4.5% of children of school-age) repeated 1 year of school, in 7 cases for indolence, in 3 for learning disabilities (LD)/health problems, and in 2 for family problems. Eleven of these children were born before maternal diagnosis.

Twelve children (4.0%; 4 males, 8 females; mean age of 12.2 years at the time of interview) were affected by AD. Children’s and maternal characteristics are shown in Table 1.

**Table 1:** Characteristics of children with and without autoimmune diseases. Autoimmune diseases were as follows: 1 juvenile idiopathic arthritis, 1 diabetes mellitus type 1, 1 autoimmune thyroiditis, 1 recurrent fever, and 8 celiac disease (CD). Among the 8 children with CD, 3 had a mother affected by CD and 2 had affected family members from the paternal side. The HLA typing was performed in 3 children and their parents: the DQ2/DQ8 haplotype was found in 2 mothers affected by CD and in one asymptomatic father

	Children with autoimmune diseases (*n* = 12)	Children without autoimmune diseases (*n* = 287)	*p*-value
Characteristics of children			
Sex (male)	4 (33.3%)	162 (56.4%)	0.2437
Child’s age at the time of interview (years, mean ± SD)	12.2 ± 6.94	17.3 ± 9.78	0.0742
Overall preterm birth (≤ 37 weeks)	4 (33.3%)	52 (18.1%)	0.2445
Late preterm birth (34.1–36.6 weeks)	3 (25.0%)	36 (12.5%)	0.7359
Early preterm birth (≤ 34 weeks)	1 (8.3%)	16 (5.6%)	0.3975
Mean weight at birth—male (kg, mean ± SD)	2.984 ± 0.469	3.277 ± 0.493	0.0439
Mean weight at birth—female (kg, mean ± SD)	2.975 ± 0.457	3.127 ± 0.515	0.3148
Maternal diagnosis			
Children born from CA mothers	3 (25%)	103 (35.8%)	
CA diagnosed before the pregnancy	1 (33.3%)	23 (22.3%)	> 0.9999
CA diagnosed after the pregnancy	2 (66.67%)	80 (77.6)	
Children born from CTD mothers	9 (75%)	184 (64.12%)	
CTD diagnosed before the pregnancy	4 (44.4%)	55 (29.8%)	0.4594
CTD diagnosed after the pregnancy	5 (55.6%)	129 (70.2%)	

Eleven children (3.7%; 7 males, 4 females, mean age of 11.9 years at the time of interview) had a certified ND and had been diagnosed as learning disabilities (LD) such as dyslexia, dyscalculia, and dysgraphia (*n* = 6); attention deficiency and hyperactivity disorder (ADHD) (*n* = 2); autism spectrum disorder (ASD) (*n* = 1); ADHD + LD; and 1 “slow learner” (a girl who was born at term but small for gestational age) (*n* = 1).

Table 2 reports on children’s and maternal characteristics with regard to foetal exposure to autoantibodies and drugs, as factors potentially impacting on the foetal neurodevelopment.

**Table 2:** Children with and without neurodevelopmental disorders: characteristics at birth, maternal diagnosis, exposure in utero to autoantibodies, and/or anti-rheumatic or anti-thrombotic drugs. As autoantibodies may be positive many years prior to the onset of symptoms and clinical diagnosis, [10] we performed an additional analysis by considering as exposed in utero to autoantibodies those children who were born within 5 years of maternal diagnosis of RD

	Children with ND (*n* = 11)	Children without ND (*n* = 288)	*p*-value
Characteristics of children			
Sex (male)	7 (63.6%)	148 (51.4%)	0.5455
Child’s age at the time of interview (years, mean ± SD)	11.9 ± 4.35	17.1 ± 9.86	0.0842
Overall preterm birth (≤ 37 weeks)	3 (27.2%)	53 (18.4%)	0.4375
Late preterm birth (34.1–36.6 weeks)	1 (9%)	38 (13.1%)	0.7264
Early preterm birth (≤ 34 weeks)	2 (18.1%)	15 (5.2%)	0.3426
Mean weight at birth—male (kg, mean ± SD)	3.0393 ± 0.562	3.29 ± 0.475	0.0890
Mean weight at birth—female (kg, mean ± SD)	2.8175 ± 0.517	3.1349 ± 0.513	0.0451
Maternal diagnosis			
Children born from CA mothers	2 (18.2%)	104 (36.1%)	0.3387
Pregnancy after the diagnosis of CA	0 (0%)	27 (26.0%)	> 0.9999
Pregnancy before the diagnosis of CA	2 (100%)	77 (74.0%)	
Children born from CTD mothers	9 (81.2%)	184 (63.9%)	
Pregnancy before the diagnosis of CTD	5 (55.6%)	56 (30.4%)	0.1443
Pregnancy before the diagnosis of CTD	4 (44.4%)	128 (69.6%)	
In utero exposure to drugs			
Exposure to at least one drug	5 (45.5%)	69 (24.0%)	0.1477
Oral prednisone/methylprednisolone (CS)	5 (45.5%)	49 (17.0%)	0.0311
Azathioprine (AZA)	1 (9.1%)	2 (0.7%)	0.1067
Cyclosporin-A (CyA)	0 (0%)	1 (0.3%)	> 0.9999
Intravenous immunoglobulins (IVIG)	0 (0%)	2 (0.7%)	> 0.9999
Hydroxychloroquine (HCQ)	0 (0%)	26 (9.0%)	0.6073
Heparin (LMWH)	0 (0%)	14 (4.9%)	> 0.9999
Low-dose acetylsalicylic acid (LDA)	2 (18.2%)	26 (9.0%)	0.2751
Leflunomide (LEF)	0 (0%)	0 (0%)	> 0.9999
Sulfasalazine (SSZ)	0 (0%)	1 (0.3%)	> 0.9999
Methotrexate (MTX)	0 (0%)	1 (0.3%)	> 0.9999
In utero exposure to autoantibodies^a^			
Exposure to at least one autoantibody	5 (100.0%)	59 (71.1%)	0.3172
Antiphospholipid antibodies (at least one positive test)	2 (40.0%)	19 (22.9%)	0.5891
Anti-cardiolipin antibodies (aCL)	2 (40.0%)	16 (19.3%)	0.2698
Anti-Beta2glycoprotein I antibodies (aB2GPI)	0 (0.0%)	13 (15.7%)	> 0.9999
Lupus anticoagulant (LA)	0 (0.0%)	9 (10.8%)	> 0.9999
Anti-Ro/SS-A	2 (40.0%)	25 (30.1%)	0.6403
Anti-La/SS-B	1 (20.0%)	9 (10.8%)	0.4611
Anti-nuclear antibodies (ANA)	4 (80.0%)	52 (62.7%)	0.6490
Anti-dsDNA antibodies	3 (60.0%)	16 (19.3%)	0.0652
Other autoantibodies	0 (0.0%)	10 (12.0%)	> 0.9999
In utero exposure to autoantibodies^b^			
Exposure to at least one autoantibody	5 (100.0%)	92 (67.6%)	0.7161
Antiphospholipid antibodies (at least one positive test)	2 (40.0%)	24 (17.6%)	0.6356
Anti-cardiolipin antibodies	2 (40.0%)	21 (15.4%)	0.6140
Anti-Beta2glycoprotein I antibodies	0 (0.0%)	16 (11.8%)	0.5980
Lupus anticoagulant	0 (0.0%)	15 (11.0%)	> 0.9999
Anti-Ro/SS-A	2 (40.0%)	36 (26.5%)	> 0.9999
Anti-La/SS-B	1 (20.0%)	12 (8.8%)	0.5402
Anti-nuclear antibodies	5 (100.0%)	82 (60.3%)	> 0.9999
Anti-dsDNA antibodies	3 (60.0%)	23 (16.9%)	0.1565
Other autoantibodies	0 (0.0%)	22 (16.2%)	0.6083

^a^This section considers all the live-birth pregnancies occurred after the diagnosis of RD (88 children, including 5 children with ND and 83 children without ND)

^b^This section considers all the live-birth pregnancies occurred in the 5 years prior to the diagnosis of RD and all the pregnancies occurred afterwards (144 children, including 8 children with ND and 136 children without ND)

Details about the children with AD and ND and their mothers are reported in Table 3.

**Table 3:** Children affected by autoimmune diseases (AD) or neurodevelopmental disorders (ND): characteristics of mothers and offspring.

	Sex	Age at the time of the interview	Disease	Birth before/after maternal disease onset	Gestational week at birth	Maternal disease	In utero exposure to maternal autoantibodies	In utero exposure to drugs
Autoimmune diseases (AD)								
1	M	25	Type 1 diabetes	Before	32	RA	NA	No
2	F	26	Juvenile idiopathic arthritis	Before	41	RA	NA	No
3	F	5	Celiac disease	After	38	RA	No	HCQ
4	F	14	Celiac disease	Before	38	UCTD	NA	No
5	F	13	Autoimmune hypothyroidism	Before	41	DM/PM	NA	No
6	M	9	Celiac disease	Before	36	SS	NA	No
7	F	11	Celiac disease	Before	39	UCTD	NA	No
8	F	8	Celiac disease	Before	38	SLE	ANA, a-dsDNA, aCL, aB2GPI, anti-Ro/SSA*	No
9	M	4	Celiac disease	After	36	UCTD	Anti-Ro/SSA, LA, ANA	CS, IvIg
10	M	8	Celiac disease	After	41	SLE	ANA, a-dsDNA	HCQ, LDA
11	F	10	Celiac disease	After	35	SSc	ANA, anti-Scl70, anti-RNP	CS, LMWH
12	F	13	Recurrent fever	After	36	SLE	Anti-Ro/SSA, LA, ANA, a-dsDNA	CS, HCQ
Neurodevelopmental disorders (ND)								
1	M	17	ADHD + LD	Before	37	RA	NA	No
2	F	16	Slow learner	Before	38	RA	NA	No
3	M	12	ADHD + ASD	Before	28	SSc	ANA, a-TPO, a-TG IgA**	CS, LDA, LMWH**
4	M	17	LD (dyslexia)	Before	39	SSc	NA	No
5-	F	18	LD (dyscalculia)	Before	40	SSc	NA	No
6	F	7	ADHD	Before	38	SLE	ANA*	No
7	F	10	LD (dyslexia, dyscalculia)	After	40	SLE	Anti-Ro/SSA, aCL	CS
8	M	7	ADHD	After	36	SLE	ANA, aCL	CS, AZA, LDA
9	M	8	ADHD + LD (dyslexia, dyscalculia)	After	42	SLE	ANA, a-dsDNA	No
10	F	8	LD (dysnomia)	After	34	SLE	ANA, a-dsDNA	CS
11	M	12	LD (dyslexia)	After	38	SLE	Data not available	CS

*aB2GPI* anti-beta2glycoprotein I antibodies, *aCL* anti-cardiolipin antibodies, *ADHD* attention deficit hyperactivity disorder, *a-dsDNA* anti-dsDNA antibodies, *ANA* anti-nuclear antibodies, *anti-RNP* anti-ribonucleoprotein antibodies, *ASD* autism spectrum disorders, *a-TG* anti-transglutaminase antibodies, *a-TPO* anti-thyroperoxidase antibodies(, *AZA* azathioprine, *CS* oral prednisone/methylprednisolone, *DM/PM* dermato-polymyositis, *HCQ* hydroxychloroquine, *IVIG* intravenous immunoglobulins, *LA* lupus anticoagulant, *LD* learning disabilities, *LDA* low-dose acetylsalicylic acid, *LMWH* low molecular weight heparin, *NA* not applicable, *RA* rheumatoid arthritis, *SLE* systemic lupus erythematosus, *SS* Sjögren’s syndrome, *SSc* systemic sclerosis, *UCTD* undifferentiated connective tissue disease

*As the pregnancy occurred within the 5 years prior to diagnosis, it is possible to assume that autoantibodies were already present during pregnancy

**This patient was tested for autoantibodies because of infertility and was treated by obstetric indication for favouring better outcomes of a spontaneous pregnancy

## Discussion

This study aimed at assessing the long-term outcome of children born to mothers with a definite diagnosis of RD. To our knowledge, this is the only study comprising young adults (mean age of 17.1 years), i.e. the age of possible onset of AD.

We found that AD, mainly organ-specific, were present in 3.8% of the offspring, while a systemic disease was present in 2 cases (0.6%). This figure can be considered as comparable with the recently published study of children born to mothers with SLE in Canada [12], in which the frequency of AD was 1.1%, while it was 0.5% in the control group. Interestingly, no cases of celiac disease (CD) were reported in SLE offspring and only one case was found in the controls. In our cohort, 8 out of the 12 children with AD were affected by CD. This difference highlights that variability in genetic heritage and exposome may account for different frequency of specific AD in different populations [11]. The frequency of CD found in our study (2.5%) does not seem to be significantly higher than that reported more than 10 years ago in the general paediatric population (0.33–1.25%) [13]. The increasing disease awareness and diagnostic sensitivity of AD and in particular of CD, which lately seems to have become “epidemic,” [14] cannot support a hint toward an increased risk of CD in our cohort. As a matter of fact, one fourth of the affected children had family members with CD on the paternal side (data not shown).

The observed frequency of ND was 3.7%. Despite absolute numbers are rather low, there was a clustering in children born to mothers with CTD (81%), particularly after maternal diagnosis (66.6%). This observation is in line with the reports of the last 30 years suggesting that children born to mothers with SLE seem to be more prone to ND [9]. Children with ND were more frequently exposed in utero to corticosteroids and maternal anti-dsDNA antibodies. However, the placental enzymes physiologically inactivate prednisone/methylprednisolone and only about 10% can reach the foetus limiting their pathogenic potential. On the other hand, the trend toward an increased frequency of exposure to maternal anti-dsDNA is consistent with an animal model showing that a subset of anti-dsDNA called anti-N-methyl-D-aspartate-receptor antibodies were able to induce foetal neuronal apoptosis and ND in the offspring [6]. Both corticosteroids and anti-dsDNA can point at active maternal disease during pregnancy, which is in turn a risk factor for premature birth. ND are multifactorial in origin; therefore, the in utero environment is just one of the risk factors. Prematurity can be indeed a major risk factor for ND. Although there was no statistically significant difference in the rate of pre-term birth between children with and without ND, the frequency of pre-term birth in our cohort (18.4%) was higher than that of the Italian general obstetric population (12%) [15]. The causes of pre-term birth were not collected in our survey. However, it is possible that some of these pre-term births were iatrogenic, as in the past women with RD were not let to deliver at full term because of the fear of maternal disease flares.

LD were the most common ND in our cohort with a frequency of 1.8%, similar to that of 2.5–3.5% described in Italian school-children [16]. The occurrence of LD was not associated with the in utero exposure to maternal antiphospholipid antibodies (aPL), but none of the patients carried a triple aPL positivity, which was the common feature of children with LD, as recently reported [17].

Only one case of ASD was found in our cohort. The possible association between ASD and maternal RD should not be emphasized in our opinion as no solid causative explanation has been elaborated yet. The fact that ASD were found in children born to mothers with different RD such as RA, [7, 18] SLE [8], and APS [19] may reflect their sporadic occurrence rather than a biologically supported association.

We acknowledge that this study has some limitations: (i) it was not designed as a case-control study; however, it carries the relevant information for future parents that the chance of occurrence AD and NP in their offspring is low [6]; (ii) the survey did not collect information about Caesarean section, linked to an increased risk of non-rheumatic autoimmune diseases [12]; and (iii) a national cohort does not allow to generalize the counselling to women with a different ethnic background.

There are also some strengths in this study: (a) it provides data regarding the long-term outcome of children born to women with RD other than RA, SLE, APS; (b) the enrolment of consecutive patients attending the out-patient clinics of 24 centres across the country during the same time period allowed random inclusion of children; (c) the direct contact with the patients allowed us to retrieve validated data about children’s outcomes and exposures during pregnancy, while administrative sources can misclassify the diagnosis and do not allow precise information about drugs [20]; and (d) the mean age of 17 years accounts for the longest follow-up of children born to mother with RD available in the literature, allowing a better assessment of AD as their onset is usually during adulthood.

In conclusion, according to our data, a link between maternal disease (particularly CTD) and ND cannot be excluded, suggesting the need of attention by the healthcare providers to explore any possible concern of the mother. However, our study found a low frequency of AD and ND in a large randomly selected cohort of children born to mothers with RD. This is a valuable information for the counselling of patients of childbearing age that was lacking in clinical practice.

## Key Messages

- Knowledge about the long-term outcome of children born to mothers with rheumatic diseases (RD) is an unmet need in the counselling about reproductive issues.
- Some studies suggested that the offspring of women with rheumatoid arthritis and systemic lupus erythematosus may be at increased risk of autoimmune diseases (AD) and neurodevelopmental disorders (ND).
- We collected information about 299 randomly selected individuals born to mothers with RD with a mean age of 17 years at the time of the survey, in 24 rheumatology centres across Italy.
- Absolute numbers of offspring affected by AD and ND were low; a possible link between maternal connective tissue diseases and ND needs to be further investigated, although we did not find any association with the in utero exposure to maternal autoantibodies and anti-rheumatic drugs.
- Concerns regarding the health outcomes of the offspring might not be an issue discouraging women with RD from having children.

## Data Availability

Data are available on reasonable request.
